# Phytochemical Enrichment of Carrot Seed Extracts by Ethanol-Modified Supercritical Fluid Extraction: Antimicrobial, Enzyme-Inhibitory, Butyrylcholinesterase Inhibition and Molecular Docking Investigations

**DOI:** 10.3390/foods15101721

**Published:** 2026-05-13

**Authors:** Husam Qanash, Sulaiman A. Alsalamah, Abdulrahman S. Bazaid, Fahad Almarshadi, Mohammed Ibrahim Alghonaim, Waleed Hakami, Amro Duhduh, Nourah M. Almimoni

**Affiliations:** 1 Department of Medical Laboratory Science, College of Applied Medical Sciences, University of Ha’il, Hail 55476, Saudi Arabia; 2 Medical and Diagnostic Research Center, University of Ha’il, Hail 55473, Saudi Arabia; 3 Department of Biology, College of Science, Imam Mohammad Ibn Saud Islamic University (IMSIU), Riyadh 11623, Saudi Arabia; 4 Public Health Department, College of Public Health and Health Informatics, University of Ha’il, Hail 55473, Saudi Arabia; 5 Department of Medical Laboratory Technology, College of Nursing and Health Sciences, Jazan University, Jazan 45142, Saudi Arabia; 6 Department of Biology, Faculty of Science, Al-Baha University, Al-Baha 65779, Saudi Arabia

**Keywords:** carrot seed extract, supercritical fluid extraction, ethanol co-solvent, antimicrobial activity, α-amylase/α-glucosidase inhibition activity, butyrylcholinesterase inhibition, molecular docking

## Abstract

This study explored the impact of ethanol as a co-solvent in supercritical fluid extraction on the recovery of bioactive compounds from carrot seeds and assessed the resulting extracts for antimicrobial, α-amylase and α-glucosidase, and butyrylcholinesterase inhibitory potential. Ethanol supplementation significantly improved extraction performance, with the yield increasing from 110 mg in the absence of ethanol to 134 mg at 5% ethanol, followed by a slight decrease to 132 mg at 10%. High-performance liquid chromatography (HPLC) revealed pronounced phytochemical enrichment at 5% ethanol, particularly for chlorogenic acid (1541.24 µg/g), gallic acid (1279.27 µg/g), and hesperetin (1513.68 µg/g), indicating enhanced recovery of phenolic and flavonoid constituents. The 5% ethanol extract demonstrated superior antimicrobial activity, producing inhibition zones of 19 mm against *Enterococcus faecalis*, 26 mm against *Klebsiella pneumoniae*, 25 mm against *Staphylococcus aureus*, and 29 mm against *Candida albicans*. Values of both minimum inhibitory concentration (MIC) and minimum bactericidal concentration (MBC) were markedly reduced, while antibiofilm activity reached 93.11% for *E. faecalis* and 91.00% for *K. pneumoniae*. The extract also exhibited potent inhibitory effects with IC_50_ values of 7.74 and 13.37 µg/mL, against α-amylase and α-glucosidase, correspondingly, as well as strong butyrylcholinesterase inhibition (IC_50_ = 2.51 µg/mL), highlighting promising α-amylase/α-glucosidase and butyrylcholinesterase inhibitory potential. Molecular docking further supported these findings, showing that chlorogenic acid bound more strongly than vanillin to OmpK36, lysosomal acid-α-glucosidase, and butyrylcholinesterase, with docking scores ranging from −6.1 to −6.9 kcal/mol. These findings identify ethanol-modified supercritical fluid extraction as a sustainable and effective green strategy for improving the recovery of carrot seed bioactives and enhancing their multifunctional in vitro biological properties. Notably, this study provides the first comprehensive evidence that 5% ethanol modification selectively enriches key phenolic constituents, including chlorogenic acid, gallic acid, and hesperetin, in carrot seed extracts, with corresponding enhancement of α-amylase, α-glucosidase, and butyrylcholinesterase inhibitory activities.

## 1. Introduction

Plant-derived bioactive compounds are naturally occurring chemical constituents that are typically present in low concentrations in plant tissues. These compounds have attracted substantial scientific attention because of their perceived safety, environmental compatibility, and beneficial effects on human health [1,2]. Hence, considerable research has been invested in the development of actual extraction methods capable of recovering these valuable constituents. Although numerous conventional extraction techniques are available, no single standardized or universally optimized method has been established for isolating bioactive compounds from plant matrices [3,4,5]. Therefore, current research is increasingly focused on innovative and sustainable strategies that can improve the efficiency and selectivity of phytochemical extraction.

*Daucus carota* L. (carrot) represents a widely cultivated biennial crop with notable importance not only for its edible root but also for its diverse phytochemical composition. It is among the most consumed vegetables worldwide and is recognized for both its nutritional and functional importance [6]. In addition to the root, carrot seeds represent an underexplored but promising a valuable reservoir of bioactive molecules, notably phenolics, carotenoids, flavonoids, glycosides, alkaloids, and volatile constituents, all of which may contribute to their biological activities [7]. In recent years, carrot seed extracts have gained increasing attention because of their broad pharmacological potential. Several studies have reported notable antioxidant and antimicrobial activities associated with these extracts [8]. For example, carrot seed oil demonstrating inhibitory activity toward pathogenic species such as *Escherichia coli* and *Staphylococcus aureus* [9]. Likewise, advanced extraction approaches such as microwave-assisted extraction have been reported to generate higher rates of phenolic and flavonoid compounds than conventional Soxhlet extraction, resulting in improved antioxidant and antifungal properties [10].

Beyond their antimicrobial potential, carrot seed extracts have also demonstrated promising therapeutic effects. Experimental studies have reported hypolipidemic activity in animal models, including reductions in total cholesterol and triglycerides [11]. Hepatoprotective effects have also been attributed to the antioxidant capacity of methanolic carrot seed extracts [11]. In addition, Ksouri et al. [12] found that ethanolic carrot seed extracts exhibited the strongest radical-scavenging activity among the tested extraction systems, revealing the impact of solvent selection on the efficiency of solvent selection in maximizing the recovery of antioxidant constituents.

Butyrylcholinesterase inhibitory properties have also been documented. Carrot seed extracts were reported to improve memory and reverse chemically induced amnesia in animal models, suggesting potential anti-Alzheimer effects through modulation of cholinesterase activity [13]. Furthermore, the phytochemical richness of carrot seeds supports their reported α-amylase/α-glucosidase inhibition, anti-inflammatory, and anticancer activities. Collectively, these findings indicate that carrot seeds are a promising natural source of multifunctional bioactive compounds with considerable pharmaceutical and nutraceutical value.

Despite these encouraging reports, the extraction of carrot seed bioactivities still relies predominantly on conventional or semi-advanced techniques, while green and highly efficient methods such as supercritical fluid extraction (SFE) remain insufficiently explored. SFE, particularly when carbon dioxide is used as the primary extraction solvent, offers several advantages, including high selectivity, minimal solvent residues, and improved preservation of thermolabile compounds [14]. The incorporation of a polar modifier, such as ethanol, can further enhance the solubility and recovery of moderately polar phytochemicals [14]. Nevertheless, the application of ethanol-modified supercritical fluid extraction to carrot seeds remains insufficiently explored. However, the application of ethanol-modified SFE to carrot seeds has received limited attention. Compared with conventional extraction techniques such as Soxhlet extraction and microwave-assisted extraction, supercritical fluid extraction offers distinct advantages for the recovery of carrot seed bioactives. Soxhlet extraction, although widely used, requires large volumes of organic solvents, prolonged extraction times, and elevated temperatures that may lead to degradation of thermolabile phenolic compounds [10]. Microwave-assisted extraction provides more rapid extraction and reduced solvent consumption, but it may cause localized overheating and generally requires an additional post-extraction filtration step [10]. In contrast, supercritical fluid extraction using carbon dioxide as the primary solvent operates at relatively low temperatures, minimizes solvent residues, enables selective extraction through adjustment of pressure and temperature, and better preserves thermolabile compounds. However, supercritical fluid extraction equipment is more expensive than conventional systems, and careful optimization of process parameters, including co-solvent concentration, remains essential for the efficient recovery of moderately polar phytochemicals [14].

In parallel, molecular docking has proven to be a strong computational mean for predicting the binding orientation and affinity of small molecules within the active sites of target proteins [15]. This approach helps clarify ligand-protein interactions and supports the identification of potentially active phytochemicals. Moreover, molecular docking complements in-vitro and in-vivo findings by providing mechanistic insight at the molecular level, thereby strengthening the interpretation of experimental bioactivity data [1].

Accordingly, the existing study designed to investigate the influence of ethanol as a co-solvent in the supercritical fluid extraction of carrot seed bioactive compounds and to evaluate the resulting extracts for antimicrobial, antibiofilm, α-amylase/α-glucosidase inhibition, and butyrylcholinesterase inhibitory activities. In addition, molecular docking analysis was conducted to explore the interaction profiles of selected phytochemicals with relevant biological targets. By addressing this research gap, the study contributes to the valorization of carrot seeds as a functional and pharmaceutical resource while advancing environmentally friendly extraction technologies.

The novelty of the present study lies in several key aspects. First, although supercritical fluid extraction has been applied to various plant materials, its systematic optimization using ethanol as a co-solvent for carrot seeds, together with comprehensive evaluation of extraction yield, phytochemical composition, and multiple in vitro biological activities, has not been previously reported. Second, unlike earlier studies that focused on a single activity, such as antimicrobial or antioxidant effects alone, the present work simultaneously evaluates antimicrobial, antibiofilm, α-amylase and α-glucosidase inhibitory, and butyrylcholinesterase inhibitory activities, thereby providing a broader functional assessment of the extract. Third, the integration of experimental bioactivity data with molecular docking analysis of identified phytochemicals offers additional mechanistic insight into the observed effects. Fourth, this study directly compares supercritical fluid extraction with and without ethanol modification, allowing quantitative assessment of the enhancement effect on individual phenolic constituents rather than only on total phenolic content. To the best of our knowledge, this is the first report demonstrating that 5% ethanol-modified supercritical fluid extraction selectively enriches chlorogenic acid, gallic acid, and hesperetin from carrot seeds, with corresponding enhancement of α-amylase, α-glucosidase, and butyrylcholinesterase inhibitory potential.

## 2. Materials and Methods

### 2.1. Procurement of Plant Sample

Seeds of *Daucus carota* L. (family Apiaceae) were obtained from the Agriculture Research Center in Saudi Arabia. The plant material was formally identified by Dr. Qanash, Department of Medical Laboratory Science, University of Ha’il. A voucher specimen (accession number: Hail-2024-072) has been deposited in the Herbarium of the Medical and Diagnostic Research Center, University of Ha’il, Hail, Saudi Arabia. The seeds were cleaned, finely ground, and stored in airtight containers until use.

### 2.2. Supercritical Fluid Extraction of Carrot Seeds

Carrot seeds were used as the plant matrix for supercritical fluid extraction [14]. Briefly, dried seeds (The 4 g sample size corresponds to the standard extraction vessel capacity of the ISCO-SFX 220 system) were fed into the extraction unit of an ISCO-SFX 220 supercritical fluid extractor (UK). Carbon dioxide (CO_2_, 98% purity, SFE grade) was used as the principal extraction solvent, whereas ethanol was functionalized as a polar co-solvent to enhance the extraction of bioactive constituents. To minimize batch-to-batch variability, seeds from multiple collection batches were pooled, thoroughly mixed, and ground as a single composite lot to ensure sample homogeneity. Three independent extraction replicates were performed for each ethanol condition, as specified in Section 2.9. The extraction process was carried out at a constant temperature of 40 °C and a pressure of 250 bar. Each extraction run consisted of two consecutive stages: a static phase and a dynamic phase. During the static phase, the system was maintained for 25 min without solvent flow to allow adequate penetration of supercritical CO_2_ into the plant matrix. This was followed by a dynamic extraction phase of 40 min, during which supercritical CO_2_ was continuously passed through the sample at a flow proportion of 3 mL/min. Ethanol was delivered using a Series II isocratic HPLC pump, while CO_2_ flow was controlled using an SFT-10 constant-pressure pump. Different ethanol concentrations were evaluated as extraction modifiers. The extraction temperature of 40 °C and the pressure of 250 bar were selected based on preliminary optimization experiments as well as previously reported conditions considered suitable for the extraction of phenolic compounds from oilseeds and other plant materials [14]. Preliminary trials examined temperatures ranging from 35 to 55 °C and pressures from 200 to 300 bar, with 40 °C and 250 bar providing the most favorable combination of extraction yield and total phenolic recovery while maintaining compound stability. These conditions are also consistent with previously reported parameters for supercritical carbon dioxide extraction of carrot seed bioactives [14]. The extraction yield was initially compared to 0%, 5%, and 10% ethanol conditions, whereas the 0% and 5% ethanol extracts were selected for further phytochemical and biological analyses. Extracts were collected in glass vials and concentrated under reduced pressure to remove residual solvents, yielding crude viscous residues.

### 2.3. Identification and Quantification of Phenolics and Flavonoids via HPLC

Analysis of phenolic and flavonoid compounds in carrot seed extracts was carried out via high-performance liquid chromatography (HPLC) using a Knauer system (Germany) equipped with a C_18_ column (4.6 mm i.d. × 150 mm, 5 µm particle size) and a UV detector set at 350 nm, according to the method described by Bazaid et al. [16]. The mobile phase comprised solvent A, which consisted of 0.15% phosphoric acid in a mixture of water: methanol (77:23, *v*/*v*; pH 2), and solvent B, composed of methanol. The elution was carried out according to the following gradient program: from 0 to 3.6 min, 100% A under isocratic conditions; from 3.6 to 30 min, a linear gradient was applied to reach 80.5% A; from 30 to 60 min, 80.5% A was maintained isocratically; from 60 to 67.2 min, a further linear gradient was used to 51.8% A; and after 67.2 min, the system was switched to 100% B.

The flow rate was maintained at 1.0 mL/min, and the injection volume was 20 µL. To improve compound identification, relative retention indices were calculated to compensate for possible retention time variation. Peaks detected in the chromatograms were identified by comparing their relative retention indices with those of co-injected authentic standards. The relative abundance of each flavonoid and phenolic compound was quantified from the corresponding chromatographic peak areas. Quantification was performed using external standard calibration curves for each authentic standard (purity ≥ 98%, Sigma-Aldrich, St. Louis, MO, USA). Standard solutions were prepared at concentrations ranging from 1 to 500 µg/mL, and calibration curves were constructed with correlation coefficients (R^2^) greater than 0.995 for all compounds. Quality-control measures included: (1) injection of standard mixtures after every 10 sample injections to monitor retention-time stability, (2) duplicate injections of each extract sample, and (3) spike-recovery tests performed by adding known amounts of standards to selected samples, yielding recovery rates ranging from 92% to 106% for all quantified compounds. Relative retention indices were also calculated to compensate for possible retention-time variation, as described in the method section.

### 2.4. Agar Well Diffusion Assay for Antimicrobial Efficacy

The antimicrobial efficacy of carrot seed extracts was assessed employing the agar well diffusion approach with minor modifications. The tested microorganisms included the *Enterococcus faecalis* (ATCC 29212), *Staphylococcus aureus* (ATCC 6538), *Escherichia coli* (ATCC 8739) and *Klebsiella pneumoniae* (ATCC 13883), in addition to fungal strains including *Candida albicans* (ATCC 10221) and *Aspergillus* spp. A volume of 20 mL molten agar medium was poured into sterile Petri dishes (9 cm diameter) and maintained at approximately 45 °C. Microbial inocula (100 µL) were spread evenly over the agar surface using standardized suspensions. Bacterial inocula (10^8^ CFU/mL) were cultured on nutrient agar, yeast cells (10^6^ CFU/mL) on Mueller-Hinton agar, and filamentous fungal spores (10^4^ spores/mL) on Sabouraud dextrose agar. After solidification and drying, via a sterile cork borer, wells of 6 mm diameter were created, and 50 µL of carrot seed extract was introduced into each well. The prepared plates were subjected to incubation at 37 °C for 24–48 h for bacterial and yeast strains and at 25 °C for 72 h for filamentous fungi. Gentamicin was used as the standard antibacterial agent, and an antifungal drug was used as the positive control for fungal strains. The extract concentration applied to each well was 50 mg/mL, with 50 µL of the extract solution prepared in 5% DMSO. Solvent control wells contain 5% DMSO, whereas sterility control wells contain sterile water. We have also clarified that the final dimethyl sulfoxide concentration in the assay system was ≤1%, and control experiments confirmed that this concentration did not affect microbial growth. Antimicrobial activity was expressed as the diameter of the inhibition zone formed around each well [17]. In addition, the minimum inhibitory concentration was determined using the standard broth microdilution method over a concentration range of 0.98–1000 µg/mL. Serial dilutions of the extract were prepared in suitable growth media and inoculated with standardized microbial suspensions. Following incubation, the MIC was defined as the lowest concentration that completely suppressed visible microbial growth. For determination of the minimum bactericidal concentration (MBC), samples from MIC wells showing no visible growth were sub-cultured onto fresh agar plates. After further incubation, the MBC was identified as the lowest concentration at which no bacterial colonies developed. The minimum fungicidal concentration (MFC) was assessed in a similar manner by transferring aliquots from non-turbid MIC wells onto fungal growth media and recording the lowest concentration that inhibited visible fungal colony formation.

### 2.5. Biofilm Inhibition Assay

The antibiofilm efficacy of carrot seed extracts was estimated using 96-well flat-bottom polystyrene microplates according to a modified previously published method [18]. Briefly, 300 µL of freshly prepared Trypticase Soy Yeast broth containing approximately 10^6^ CFU/mL of the test organism was dispensed into each well. Sublethal extract concentrations corresponding to 75%, 50%, and 25% of the minimum bactericidal concentration were tested. The extracts were added simultaneously with the bacterial inoculum at the beginning of the 48 h incubation period, allowing us to evaluate inhibition of biofilm formation rather than eradication of preformed mature biofilms. We have also clarified that the observed reduction in biofilm biomass is discussed as antibiofilm activity under sublethal conditions, rather than as a direct measure of mature biofilm destruction. For each extract concentration, a blank well containing the extract in sterile medium without bacteria was included for each extract concentration, and the corresponding background absorbance was subtracted. The sub-inhibitory concentrations of 25%, 50%, and 75% of the minimum bactericidal concentration were selected based on established protocols for evaluating antibiofilm activity without causing complete inhibition of microbial growth [18]. This concentration range enables assessment of specific antibiofilm effects rather than effects that merely reflect growth inhibition. Preliminary experiments confirmed that these concentrations permitted microbial survival while suppressing biofilm formation. Specifically, 25% of the minimum bactericidal concentration represented a low sub-inhibitory level, 50% represented an intermediate level, and 75% represented a high sub-inhibitory level, thereby allowing evaluation of dose-dependent antibiofilm activity, as previously described [18]. Control wells included medium alone, bacterial culture without extract, and methanol-treated wells. The microplates were incubated at 37 °C for 48 h to allow biofilm formation. The supernatant was then carefully removed, and the wells were gently washed with sterile distilled water to remove planktonic cells. After air-drying for 30 min, the adherent biofilms were stained with 0.1% crystal violet for 15 min at room temperature. Excess stains were removed by washing the wells three times with sterile distilled water. To quantify biofilm biomass, 250 µL of 95% ethanol was added to each well to solubilize the bound dye. After 15 min, absorbance was quantified at 570 nm using a reader of microplate. The extent of biofilm suppression was quantified as a percentage was calculated using the appropriate standard equation.

$$\mathrm{Inhibition}\;\mathrm{of}\;\mathrm{Biofilm}\;(\%)=\left[1-\frac{\left({\mathrm{Abs}}_{\mathrm{Sample}}-{\mathrm{Abs}}_{\mathrm{Blank}}\right)}{\left({\mathrm{Abs}}_{\mathrm{Control}}-{\mathrm{Abs}}_{\mathrm{Blank}}\right)}\right]\times 100$$

### 2.6. In Vitro α-Amylase and α-Glucosidase Inhibitory Activity of Carrot Seed Extracts

The in vitro α-amylase and α-glucosidase inhibitory potential of carrot seed extracts was assessed by measuring their inhibitory activity against two key carbohydrate-hydrolyzing enzymes, α-glucosidase and α-amylase, which are directly involved in postprandial glucose regulation. For inhibition assay of α-glucosidase, 40 µL of carrot seed extract was blended with 8 µL of α-glucosidase solution (1 U/mL) and incubated at 35 °C for 18 min. Afterward, 125 µL of 0.1 M phosphate buffer (pH 6.8) was mixed in, and the reaction was triggered by introducing 20 µL of pNPG (1 M). After 30 min of incubation, the reaction was terminated by adding 50 µL of 0.1 N sodium carbonate, and absorbance was measured at 405 nm. For the α-amylase inhibition assay, the extract was dissolved in 10% DMSO and diluted with phosphate buffer containing 0.006 M NaCl and 0.02 M NaH_2_PO_4_/Na_2_HPO_4_ (pH 6.9) to obtain concentrations ranging from 1.95 to 1000 µg/mL. A 200 µL reaction mixture containing the extract and α-amylase (2 U/mL) was incubated at 30 °C for 10 min. Then, 1% starch solution (200 µL) was added and incubated for 3 min. The reaction stopped using 200 µL DNSA reagent and heated at 90 °C for 10 min. After cooling, the volume was adjusted to 5 mL with distilled water, and absorbance was read at 540 nm. For both essays, controls included enzyme activity without extract, representing 100% enzyme activity, and blanks without enzyme. Sample blanks containing the extract without enzyme were used to correct for background absorbance at each assay wavelength, namely 405 nm for α-glucosidase and 540 nm for α-amylase [19]. The percentage inhibition was calculated using the standard equation, and IC_50_ values were determined from dose-response curves plotting extract concentration against percentage inhibition.

$$\mathrm{Inhibition}\;(\%)=\frac{{\mathrm{Abs}}_{\mathrm{Control}}-{\mathrm{Abs}}_{\mathrm{Sample}}}{{\mathrm{Abs}}_{\mathrm{Control}}}\times 100$$

IC_50_ values were derived from dose–response curves constructed by plotting extract concentration against the percentage of inhibition. This combined approach provides a comprehensive assessment of the ability of carrot seed extracts to suppress carbohydrate-hydrolyzing enzymes and highlights their potential as natural α-amylase/α-glucosidase inhibition agents.

### 2.7. Butyrylcholinesterase Inhibition Assay and IC_50_ Determination

The inhibitory effect of carrot seed extracts on butyrylcholinesterase activity was evaluated using a modified Ellman colorimetric method [20]. All reagents and buffers were freshly prepared before analysis. S-butyrylthiocholine iodide (BTChI, 0.022 M) was prepared in distilled water as the substrate solution. The enzyme solution (0.44 U/mL) was prepared in phosphate buffer (pH 8.0), and Ellman’s reagent, 5,5′-dithiobis-(2-nitrobenzoic acid) (DTNB), was freshly prepared. The carrot seed extract was initially dissolved in DMSO and then diluted with distilled water to generate a series of concentrations for dose-response analysis. For the assay, 200 µL of phosphate buffer, 5 µL of BChE enzyme solution, 5 µL of DTNB, and 5 µL of the extract solution were added to each well of a microplate and incubated at 30 °C for 15 min. The reaction was initiated by adding 5 µL of BTChI substrate solution. Blanks containing the extract without substrate were included for each tested concentration. Absorbance was monitored at 410 nm using a microplate reader at 45 s intervals under controlled temperature conditions. The percentage of BChE enzyme inhibition was estimated using the equation shown below:

$$Butyrylcholinesterase\;inhibition\left(\%\right)=100-[\frac{Rate\;of\;reaction\;with\;extract}{Rate\;of\;reaction\;without\;extract}\times 100]$$

To determine the IC_50_, the assay was conducted using a range of extract concentrations. A dose-response curve was then generated by plotting the percentage inhibition against the logarithm of the extract concentration.

Rivastigmine was selected as the positive control because it is a dual inhibitor of both acetylcholinesterase and butyrylcholinesterase, making it particularly relevant for butyrylcholinesterase-specific inhibition assays [20]. In contrast, donepezil primarily inhibits acetylcholinesterase and exhibits comparatively limited butyrylcholinesterase activity. Therefore, rivastigmine was considered the more appropriate reference compound for the present assay and is also widely used as a standard in butyrylcholinesterase inhibition studies [20].

### 2.8. Molecular Docking Analysis

Crystal structures of the chosen target proteins were downloaded from the Protein Data Bank: OmpK36 (PDB ID: 5O79), lysosomal acid-α-glucosidase (PDB ID: 5NN5), and butyrylcholinesterase (PDB ID: 4BDS). Using MOE software (MOE 2024.06) (Chemical Computing Group, Montreal, QC, Canada), protein structures were prepared by eliminating water molecules, adding hydrogen atoms, assigning correct bond orders, and performing energy minimization with the AMBER10 force field: EHT force field. The structures of chlorogenic acid and vanillin were obtained from PubChem (CID_1794427 and CID_1183, respectively) and imported into MOE. The ligands were protonated, energy-minimized, and optimized using the MMFF94x force field. The Site Finder module in MOE 2019 was employed to identify the active binding sites of the target proteins. The predicted binding pockets were defined through the generation of dummy atoms. Docking calculations utilized the Triangle Matcher algorithm for conformational placement and the London dG score for initial evaluation. The top poses were further refined using the GBVI/WSA dG scoring function. For each ligand, multiple conformations were generated, and the best five poses were selected on the basis of binding energy and interaction profile. Ligand-protein interactions were analyzed using MOE visualization tools, with particular emphasis on hydrogen bonding, hydrophobic contacts, and π-interactions.

### 2.9. Statistical Analysis

All experiments were performed using three independent extraction replicates, and each assay was conducted on each replicate extract. All results are expressed as mean ± standard deviation (SD) of three independent experiments. Statistical significance among groups was evaluated using one-way analysis of variance (ANOVA). A *p*-value of ≤0.05 was considered statistically significant.

## 3. Results and Discussion

### 3.1. Extraction of Yield and Phytochemical Profiling of Carrot Seed Extracts

The extraction yield of carrot seed extracts was clearly influenced by the presence of ethanol as a co-solvent. Under the fixed supercritical fluid extraction conditions, the extract yield increased from 110 mg in the absence of ethanol to 134 mg at 5% ethanol, and then slightly decreased to 132 mg at 10% ethanol (Table 1). This trend indicates that the addition of a moderate amount of ethanol improved the solubility and recovery of bioactive constituents. A similar enhancement effect has been reported for hemp seed oil, where ethanol-modified supercritical CO_2_ extraction increased the recovery of phenolic compounds and improved the abundance of bioactive constituents [21]. These observations support the present findings and confirm that ethanol acts as an effective modifier that improves extraction efficiency during SFE.

**Figure 1:**
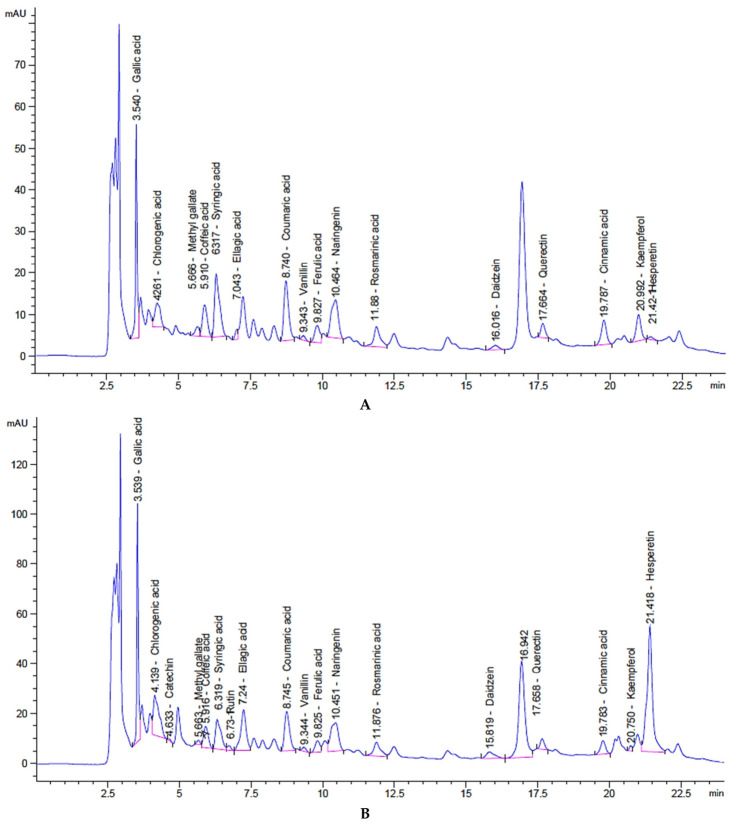
HPLC chromatographic profiles of phenolic compounds in carrot seed extracts obtained by supercritical CO_2_ extraction using (**A**) 0% ethanol and (**B**) 5% ethanol as a co-solvent.

HPLC analysis further demonstrated that the use of ethanol substantially altered the phytochemical profile of carrot seed extracts. The extract obtained with 5% ethanol contained markedly elevated levels of several phenolic and flavonoid constituents than the extract obtained without ethanol (Table 2 and Figure 1A,B). In particular, gallic acid and chlorogenic acid were enriched at 5% ethanol, reaching 1279.27 and 1541.24 µg/g, respectively, compared with 706.91 and 1464.18 µg/g in the extract prepared without ethanol. Likewise, flavonoids such as rutin and hesperetin, which were absent or present at very low levels in the extract without ethanol, were significantly increased in the 5% ethanol extract. Other compounds, including ellagic acid, naringenin, and daidzein, also showed higher concentrations under the ethanol-modified condition.

These results indicate that 5% ethanol is particularly effective in enhancing the recovery of both phenolic acids and flavonoids from carrot seeds. The improvement can be explained by the ability of ethanol in raising the polarity of supercritical CO_2_ and facilitating disruption of plant cellular structures, thereby promoting the release of intracellular metabolites. Consistent results have been reported in various plant matrices. For instance, Larocca et al. [22] verified that ethanol significantly enhanced the recovery of bioactive compounds in supercritical extraction systems. Likewise, Allay et al. [21] found that increasing ethanol concentration in SFE notably enhanced total phenolic content, confirming the strong effect of ethanol as a co-solvent. Bhatnagar et al. [23] also attributed increased extraction yields to the higher density of the CO_2_-ethanol mixture and the ability of ethanol to weaken plant cell walls. By contrast, excessively high ethanol levels may reduce the recovery of non-polar lipid fractions because of increased solvent polarity [24]. Comparable enhancement effects have also been reported for plant materials such as *Clementina orogrande* peel oil [25], cacao pod husk [26], black walnut husk [27], *Castanea sativa* [28], *Eucalyptus globulus* bark [29], and *Jatropha curcas* [30]. Overall, the present findings demonstrate that ethanol-modified SFE, particularly at 5% ethanol, is a highly effective strategy for maximizing the recovery of antioxidant-rich phytochemicals from carrot seeds. The 10% ethanol extract was not further characterized because its yield was not significantly different from that of the 5% ethanol extract (*p* > 0.05), and preliminary HPLC analysis showed no further improvement in phenolic enrichment.

An interesting observation in the present study is that although 5% ethanol significantly improved extraction yield and phenolic recovery, increasing the ethanol concentration to 10% did not result in further enhancement (Table 1). Indeed, several phenolic compounds showed lower concentrations at 10% ethanol than at 5% ethanol. This trend may be explained by several factors. First, excessive ethanol may increase the polarity of the supercritical carbon dioxide mixture beyond the optimal range for the target phenolic compounds, which could promote the co-extraction of more polar interfering constituents, such as sugars, organic acids, or polar lipids, thereby reducing extraction selectivity [24]. Second, higher ethanol levels may alter the density and solvating behavior of the supercritical fluid system, potentially reducing its affinity for specific phenolic subclasses [22]. Third, elevated ethanol concentrations may facilitate the extraction of matrix components that interfere with downstream quantification or promote aggregation or precipitation of some phenolic constituents during concentration. Similar observations have been reported in other plant matrices. For example, Allay et al. [21] found that increasing ethanol concentration beyond an optimal range led to diminishing returns in total phenolic recovery from hemp seed oil, while Quispe-Fuentes et al. [25] reported that moderate ethanol levels were optimal for phenolic extraction from citrus peel, with higher concentrations reducing extraction efficiency. Collectively, these findings support the interpretation that 5% ethanol provided a more favorable balance between enhanced solubility of phenolic constituents and preservation of extraction selectivity in carrot seed extracts.

### 3.2. Antimicrobial Activity of Carrot Seed Extracts

**Figure 2:**
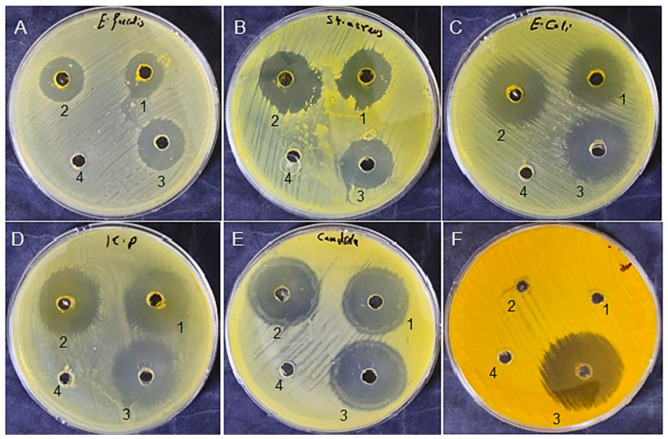
Antimicrobial effects of the tested extracts were determined by agar well diffusion against selected microorganisms. Representative plates show inhibition zones against *Enterococcus faecalis* (**A**), *Staphylococcus aureus* (**B**), *Escherichia coli* (**C**), *Klebsiella pneumoniae* (**D**), *Candida albicans* (**E**), and *Aspergillus niger* (**F**). Wells 1–4 correspond to different treatments: (1) carrot seed extracts obtained by supercritical CO_2_ extraction with 0% ethanol, (2) carrot seed extracts obtained by supercritical CO_2_ extraction with 5% ethanol, (3) positive control (Gentamicin/Nystatin), and (4) DMSO. Clear zones surrounding the wells indicate antimicrobial activity, with larger diameters reflecting stronger inhibitory effects.

Carrot seed extracts exhibited notable antimicrobial activity against all tested microorganisms, and the extract obtained with 5% ethanol generally showed superior efficacy (Table 3). The observed inhibition zones resulting from 5% ethanol extract were larger than those obtained without ethanol for most tested strains (Figure 2). For instance, inhibition against *E. faecalis* increased from 15 ± 0.5 mm to 19 ± 0.2 mm, while inhibition against *S. aureus* increased from 19 ± 0.2 mm to 25 ± 0.9 mm. Strong activity was also observed against *K. pneumoniae* and *C. albicans*. The outcomes reveal that ethanol-enhanced extraction improved the recovery of bioactive constituents responsible for antimicrobial action.

The MIC and MBC values further supported this conclusion. In several cases, the concentration required to inhibit or kill microbial cells decreased markedly in the extract prepared with 5% ethanol. For example, the MIC for *E. faecalis* decreased from 62.5 µg/mL to 15.62 µg/mL, whereas the MBC decreased from 250 µg/mL to 31.25 µg/mL. Similar improvements were observed for *S. aureus* and *E. coli*. These findings strongly suggest that ethanol-modified SFE enriched the extract with compounds possessing stronger antimicrobial potency.

Previous studies have emphasized that extraction conditions and solvent polarity play decisive roles in shaping the phytochemical profile and, consequently, the biological activity of *Daucus carota* seed extracts. Akhtar et al. [10], for example, reported that microwave-assisted extraction produced a richer phenolic and flavonoid composition than Soxhlet extraction, which translated into greater antifungal efficacy. More broadly, *Daucus carota* seeds are recognized as a rich source of phenolics, flavonoids, carotenoids, and volatile constituents that contribute to diverse pharmacological effects, including antimicrobial, antifungal, antidiabetic, anti-inflammatory, and antihypertensive activities [7]. Therefore, optimization of extraction conditions is crucial for maximizing biological activity.

The antimicrobial effects observed in the present study may be partly possibly associated with the occurrence of chlorogenic acid, gallic acid, hesperetin, and other phenolics revealed by HPLC. Chlorogenic acid has been documented as exhibiting marked antibacterial and antibiofilm properties against *Yersinia enterocolitica*, including suppression of bacterial growth and reduction of established biofilms [31]. Hesperetin has likewise demonstrated potent antibacterial activity against several pathogenic bacteria, including methicillin-resistant *Staphylococcus aureus*, by disrupting membrane integrity and promoting leakage of intracellular components [32,33]. Taken together, the present data indicate that carrot seed extracts obtained by ethanol-modified SFE possess broad-spectrum antimicrobial potential and may represent promising natural alternatives for combating microbial pathogens.

The absence of inhibitory activity against *Aspergillus niger* (Table 3, Figure 2F) is notable and may be attributed to several factors. Filamentous fungi such as *A. niger* possess thick cell walls rich in chitin and glucan, which may limit the penetration of the phenolic compounds present in the extract [4]. In addition, *A. niger* produces various efflux pumps and detoxifying enzymes that may reduce the antimicrobial efficacy of plant-derived extracts. Similar resistance of *A. niger* to plant extracts has been reported previously [4,10], suggesting that this species may be inherently less susceptible to the phenolic and flavonoid constituents enriched by ethanol-modified supercritical fluid extraction.

Unlike *Candida albicans*, which showed high susceptibility, *A. niger* was completely resistant to all tested extracts. This differential susceptibility likely reflects structural and physiological differences between yeasts and filamentous fungi. *A. niger* also produces melanin in its cell wall, which may scavenge reactive oxygen species and provide additional protection against phenolic compounds [4]. Furthermore, its complex mycelial architecture may contribute to its greater tolerance. Future studies should evaluate higher extract concentrations or combination strategies to improve anti-fungal activity against this relatively resistant species.

### 3.3. Antibiofilm Activity of Carrot Seed Extracts

The carrot seed extracts also displayed pronounced, concentration-dependent antibiofilm activity against the tested microorganisms (Table 4). In general, increasing the extract concentration from 25% to 75% of the MBC produced a substantial increase in biofilm inhibition. Moreover, the extract obtained with 5% ethanol consistently showed greater antibiofilm activity than the extract obtained without ethanol.

**Figure 3:**
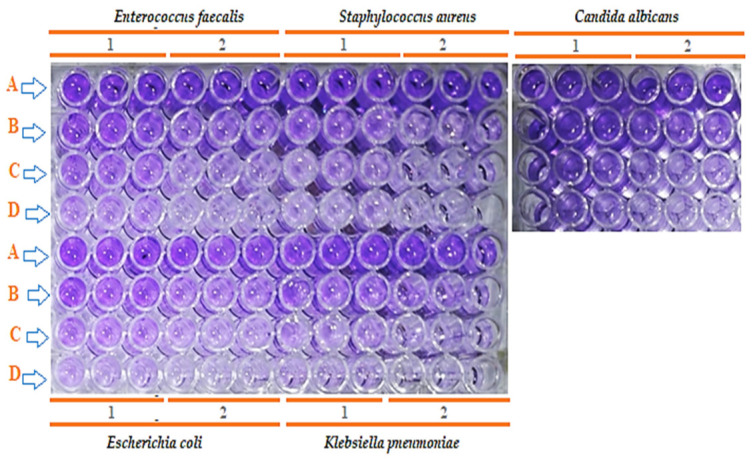
Visual assessment of the antibiofilm activity of carrot seed extracts obtained with 0% ethanol (1) and 5% ethanol (2) as extraction enhancers against *Enterococcus faecalis*, *Staphylococcus aureus*, *Escherichia coli*, *Klebsiella pneumoniae*, and *Candida albicans*, using the microtiter plate biofilm inhibition assay under different treatment conditions (rows A–D). (A) Untreated biofilm control, (B) biofilm treated with 25% of the minimum bactericidal concentration (MBC), (C) biofilm treated with 50% of the MBC, and (D) biofilm treated with 75% of the MBC of carrot seed extracts.

For example, the inhibition of *E. faecalis* biofilm formation increased from 46.60 ± 2.5% with the 0% ethanol extract to 76.90 ± 3.1% with the 5% ethanol extract at 25% MBC. Similarly, *E. coli* exhibited a marked increase in biofilm inhibition, from 38.21 ± 2.2% to 74.68 ± 3.0%. At the highest tested concentration (75% MBC), all extracts achieved strong inhibition, frequently exceeding 88%, with the 5% ethanol extract showing slightly superior performance in most cases (Figure 3).

These results suggest that ethanol-enhanced extraction improved the recovery of bioactive phytochemicals with antibiofilm properties. The lighter crystal violet staining observed in treated wells visually confirmed the reduction in biofilm biomass. Similar findings have been reported in the literature. Wangchuk et al. [34] demonstrated the antibacterial activity of carrot seed oil against *Helicobacter pylori*, highlighting the role of lipophilic constituents in microbial inhibition. Akune et al. [35] showed that solvent polarity markedly influenced the antibacterial effectiveness of carrot leaf extracts, with semi-polar solvent fractions displaying stronger inhibition against *S. aureus*. Likewise, Lathifah and Wulandari [36] reported that 70% ethanol improved extraction yield, total phenolic content, and antibacterial activity in *Daucus carota* extracts compared with 96% ethanol, emphasizing the importance of hydroalcoholic systems in recovering active compounds.

The strong antibiofilm performance observed in the present study may be linked to the phenolic compounds identified in the carrot seed extracts. Chlorogenic acid, in particular, has been reported to inhibit both bacterial growth and biofilm formation [31]. In addition, hesperetin has shown antibacterial activity through irreversible damage to bacterial cell morphology and membrane integrity [32,33]. These findings support the notion that the enhanced antibiofilm activity of the 5% ethanol extract reflects its improved phytochemical composition.

### 3.4. α-Amylase and α-Glucosidase Inhibition Activity

Carrot seed extracts prepared with 5% ethanol exhibited strong and dose-dependent inhibitory activity against both α-amylase and α-glucosidase. Enzyme inhibition increased progressively with increasing extract dose (Table 5). At the lowest tested dose (1.95 µg/mL), the extracts showed moderate inhibition, whereas at the highest concentration (1000 µg/mL), inhibition approached that of the standard inhibitor acarbose.

Importantly, the extract prepared with 5% ethanol consistently produced higher inhibitory activity than the extract obtained without ethanol. This effect was clearly reflected in the IC_50_ values. For α-amylase inhibition, the IC_50_ of the 5% ethanol extract was 7.74 µg/mL, compared with 18.56 µg/mL for the 0% ethanol extract. Similarly, for α-glucosidase inhibition, the IC_50_ values were 13.37 and 25.08 µg/mL, respectively. These findings indicate that ethanol-enhanced extraction significantly improved the in vitro α-amylase and α-glucosidase inhibitory of carrot seed extracts.

Comparable results have been reported in earlier studies where *Daucus carota* seed extracts possess inhibitory activity against carbohydrate-hydrolyzing enzymes. Tijjani and Imam [37] reported that aqueous and solvent-fractionated carrot seed extracts displayed measurable α-amylase and α-glucosidase inhibitory effects, although the potency observed in the present study was considerably greater. This superior activity may be attributed to the enrichment of phenolic and flavonoid constituents achieved through ethanol-modified SFE.

Among the compounds detected in the present work, chlorogenic acid, gallic acid, and hesperetin are especially relevant. Hassan et al. [38] testified that chlorogenic acid and gallic acid can exert synergistic α-amylase inhibitory effects, supporting their potential contribution to the observed α-amylase/α-glucosidase activity. Hesperetin has also been recognized as a hopeful natural agent for diabetes management because of its beneficial effects on glycemic control and metabolic regulation [39]. In addition, Jayaraman et al. [40] demonstrated that hesperetin administration alleviated hyperglycemia and dyslipidemia in diabetic rats by enhancing antioxidant defense systems. Taken together, the present findings highlight carrot seed extracts, especially those obtained by ethanol-modified SFE, as promising natural inhibitors of carbohydrate-digesting enzymes.

### 3.5. Butyrylcholinesterase Inhibition

Carrot seed extracts obtained at 0% and 5% ethanol exhibited concentration-dependent inhibition of butyrylcholinesterase, an enzyme strongly associated with the progression of Alzheimer’s disease (Table 6). At lower concentrations, the inhibitory effects of the extracts were modest, whereas higher concentrations resulted in substantial increases in BChE inhibition. Across the entire tested range, the extract obtained with 5% ethanol consistently showed greater inhibitory activity than the extract prepared without ethanol.

At the highest tested dose (100 µg/mL), the 5% ethanol extract achieved 89.3% inhibition, approaching the activity of the standard drug rivastigmine (93.9%). The IC_50_ values further confirmed the superior potency of the ethanol-enhanced extract, yielding values of 2.51 µg/mL and 10.50 µg/mL for the 5% and 0% ethanol extracts, respectively.

These findings suggest that ethanol-modified extraction enriches the carrot seed extract with compounds capable of interacting effectively with cholinesterase enzymes. This outcome aligns with prior research. Mohamed et al. [41] reported that ethanolic carrot seed extracts reduced oxidative stress and decreased BChE activity, while Mani et al. [13] documented memory-enhancing and anti-amnesic effects of carrot seed preparations. Atalar et al. [42] also confirmed the strong in-vitro and in-silico activity of *Daucus carota* extract against cholinesterase-related targets.

The butyrylcholinesterase inhibitory effects observed in the present investigation may be associated with the phytochemicals identified in the extract. Chlorogenic acid has been linked to neuroprotection and has been proposed as a promising therapeutic candidate in sporadic Alzheimer’s disease [43]. Hesperetin and hesperetin-rich flavonoids have also been reported to interfere with β-amyloid aggregation, one of the major pathological features of Alzheimer’s disease, thereby helping to prevent or delay neurodegeneration [44]. Thus, the strong BChE inhibition shown by the 5% ethanol extract provides additional evidence of the therapeutic promise of carrot seed bioactivities.

### 3.6. Molecular Docking Analysis of Chlorogenic Acid and Vanillin

Molecular docking analysis was performed to investigate the interaction of chlorogenic acid and vanillin with three biologically relevant targets: OmpK36 from *Klebsiella pneumoniae* (PDB ID: 5O79), human lysosomal acid-α-glucosidase (PDB ID: 5NN5), and human butyrylcholinesterase (PDB ID: 4BDS). Chlorogenic acid was selected as a representative major phenolic constituent because it was present at a relatively high concentration and has well-documented biological relevance. Vanillin, in contrast, was selected as a comparatively less abundant compound to explore whether biological activity may depend not only on abundance, but also on structural features and binding behavior. This comparative approach allowed us to examine the possibility that certain biological effects of the extract may be influenced by compounds present at lower concentrations but possessing favorable molecular interactions with the selected targets. Such an approach is often used in phytochemical studies to distinguish between quantitative abundance and qualitative bioactivity in complex mixtures. In addition, vanillin is a structurally simpler phenolic aldehyde, whereas chlorogenic acid is a more complex polyphenolic compound. Comparing these two ligands therefore provides insight into how molecular complexity and functional group diversity may influence target binding and interaction profiles. Furthermore, vanillin has previously been reported to exhibit antimicrobial and antioxidant activities [5], and its presence in *Daucus carota* extracts has also been documented [7]. Thus, the comparison between chlorogenic acid and vanillin was intended to provide a focused structure-activity perspective rather than a comprehensive screening of all detected constituents.

The docking results showed that chlorogenic acid consistently exhibited stronger binding affinity than vanillin across all targets. Specifically, chlorogenic acid achieved docking scores of up to −6.62 kcal/mol with OmpK36, −6.96 kcal/mol with glucosidase, and −6.54 kcal/mol with butyrylcholinesterase. In contrast, vanillin showed comparatively weaker interactions, exhibiting binding energies between −4.10 to −4.78 kcal/mol (Table 7, Table 8 and Table 9). Analysis of the interaction profiles further highlighted the superiority of chlorogenic acid. In the OmpK36 binding pocket, chlorogenic acid formed hydrogen bonds with ARG75 and LYS16, indicating a stable and favorable binding mode. By contrast, vanillin interacted with ASP106 and ARG37 through fewer and weaker contacts (Table 10). Within the active site of lysosomal acid-α-glucosidase, chlorogenic acid established multiple interactions, particularly with GLY123 and ILE98, suggesting improved binding stability, whereas vanillin formed a more limited interaction network (Table 11). In the case of butyrylcholinesterase, chlorogenic acid showed strong hydrogen bonding with ASN228, PRO230, and GLU238, including a notable interaction energy of −4.5 kcal/mol, whereas vanillin interacted weaker, primarily with ARG242 (Table 12).

**Figure 4:**
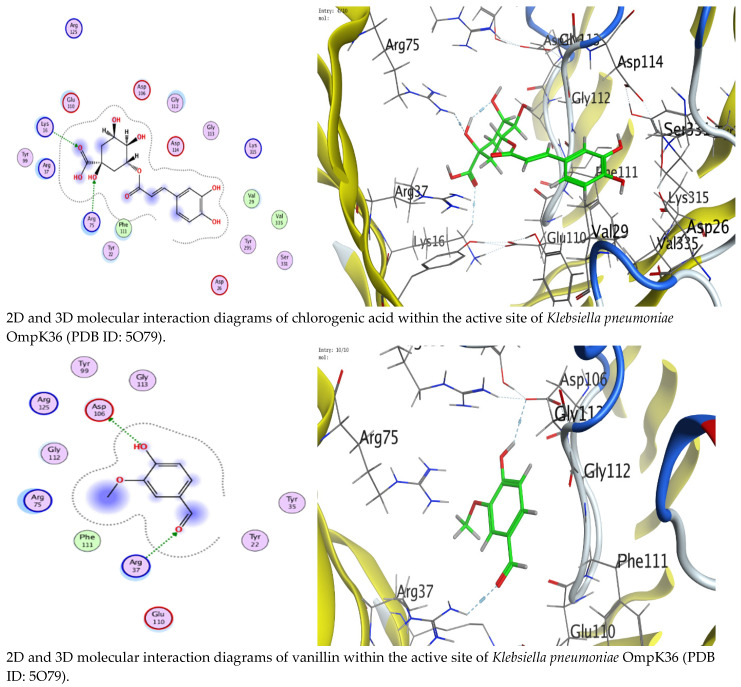

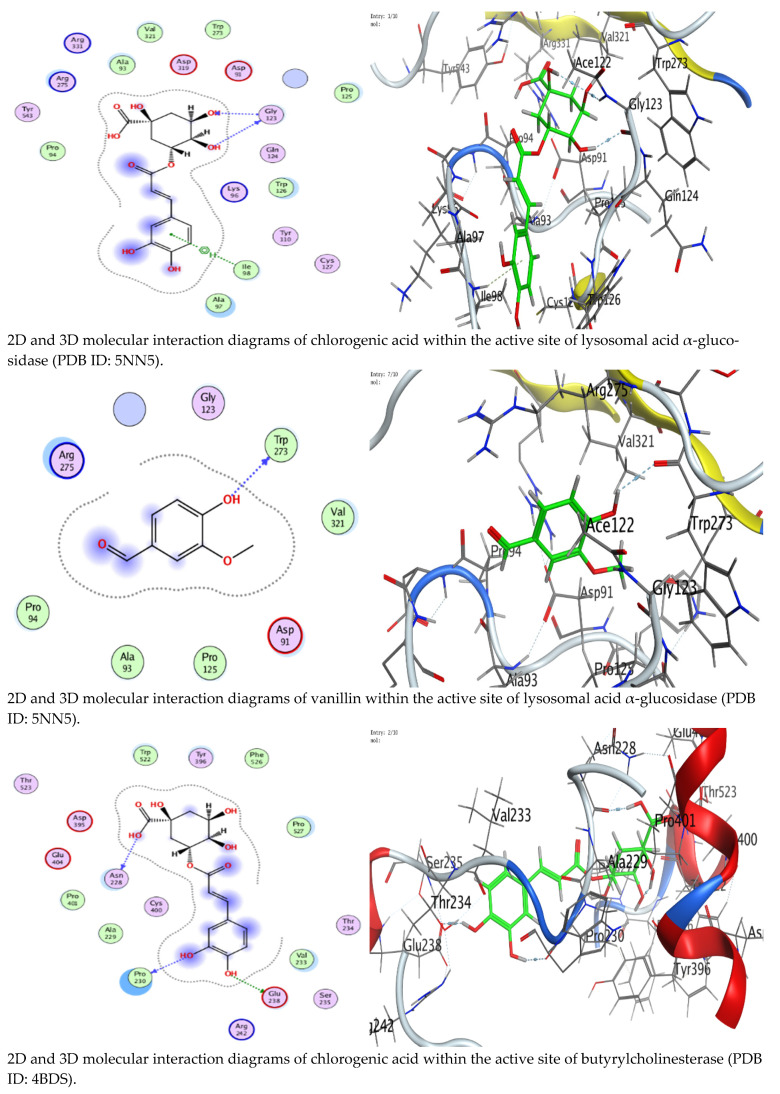

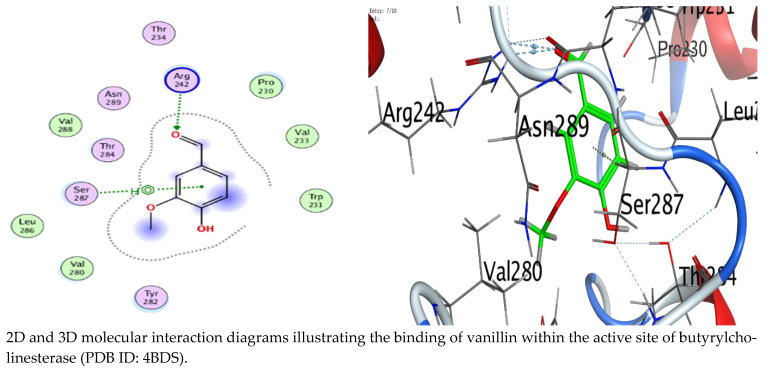
2D and 3D molecular interaction diagrams of chlorogenic acid and vanillin within the active sites of the selected target proteins, including *Klebsiella pneumoniae* OmpK36 (PDB ID: 5O79), lysosomal acid α-glucosidase (PDB ID: 5NN5), and butyrylcholinesterase (PDB ID: 4BDS).

The superior activity of chlorogenic acid is likely related to its polyphenolic structure, which provides multiple hydroxyl groups capable of forming hydrogen bonds and stabilizing the ligand-protein complex. The strong interaction with OmpK36 supports its possible contribution to the antimicrobial activity observed against *K. pneumoniae*, as OmpK36 is a porin involved in permeability and bacterial survival (Figure 4). This explanation agrees with previous literature describing the antibacterial potential of chlorogenic acid [45]. Likewise, the strong binding of chlorogenic acid to lysosomal acid-α-glucosidase aligns with the experimentally observed α-glucosidase inhibition and supports earlier reports on the role of polyphenols in modulating carbohydrate-metabolizing enzymes [46]. Finally, the favorable interaction of chlorogenic acid with butyrylcholinesterase provides a plausible molecular basis for the butyrylcholinesterase inhibitory activity detected in the existing investigation, supporting the acknowledged therapeutic value of cholinesterase inhibition in Alzheimer’s disease treatment [47].

Although vanillin is also a biologically active phytochemical, its smaller size and limited number of functional groups likely restricted its ability to form multiple stabilizing interactions, thereby explaining its weaker docking scores. Overall, the docking data strongly supports the experimental findings and identify chlorogenic acid as one of the principal contributors to the multifunctional biological activities of carrot seed extract.

Although the in vitro activities observed in this study are highly promising, several important steps are still required before any translational application can be considered. Specifically, we now state that the present work does not include in vivo efficacy studies, toxicokinetic evaluation, or cytotoxicity testing, and these limitations are now explicitly acknowledged.

For the α-amylase and α-glucosidase inhibitory activities, we note that future studies should evaluate the extract in appropriate diabetic animal models, including assessment of postprandial glucose reduction, oral glucose tolerance, glycated hemoglobin, insulin sensitivity, and pancreatic β-cell function. For the butyrylcholinesterase inhibitory activity, we indicate that validation in established neurodegeneration or memory-impairment models would be necessary, together with behavioral and biochemical analyses. We also emphasize that comprehensive safety evaluation, including cytotoxicity testing on mammalian cell lines and acute and sub-chronic toxicity studies, is essential before considering further development. In addition, we now highlight the need for bioassay-guided fractionation, compound isolation, and LC-MS/MS confirmation to verify the individual contributions of the major phytochemicals and to investigate possible synergistic effects.

### 3.7. Limitations of the Study

Despite the promising findings, this study has several limitations. First, the biological activities were evaluated mainly through in-vitro essays, which may not fully reflect in vivo efficacy, pharmacokinetics, toxicokinetics, or safety. Second, only a limited range of extraction conditions and ethanol concentrations was investigated; therefore, the absolute optimum extraction parameters may not yet have been identified. Third, the major bioactive compounds identified by high-performance liquid chromatography, including chlorogenic acid, gallic acid, and hesperetin, were not isolated, purified, or experimentally validated for their individual contributions to the observed biological activities. Although the molecular docking analysis provides mechanistic support, particularly for chlorogenic acid, experimental validation using isolated compounds and advanced chromatographic confirmation, such as preparative high-performance liquid chromatography or liquid chromatography–tandem mass spectrometry, would substantially strengthen the conclusions. Moreover, the possible synergistic or antagonistic interactions among the phytochemicals present in the crude extract remain unclear. Fourth, molecular docking was performed only for chlorogenic acid and vanillin as representative major and minor phenolic constituents, respectively, whereas other abundant compounds, such as hesperetin, gallic acid, and ellagic acid, were not included. Fifth, the study did not include cytotoxicity testing on mammalian cell lines, which is necessary to evaluate the safety profile of the extracts before any translational application. Therefore, future studies should include broader extraction optimization, compound isolation, liquid chromatography–tandem mass spectrometry confirmation, in vivo efficacy studies in appropriate animal models, cytotoxicity evaluation, and further mechanistic validation.

## 4. Conclusions

This study demonstrates that ethanol-modified supercritical fluid extraction (5% ethanol) is an effective green approach for enhancing the recovery of bioactive compounds from carrot seeds. Among the biological activities evaluated, the α-amylase and α-glucosidase inhibition potential and butyrylcholinesterase inhibition activity represent the most promising translational applications. The potent α-amylase (IC_50_ = 7.74 µg/mL) and α-glucosidase (IC_50_ = 13.37 µg/mL) inhibitory activities, combined with the strong BChE inhibition (IC_50_ = 2.51 µg/mL), position these carrot seed extracts as competitive natural alternatives to conventional therapeutics. While the antimicrobial and antibiofilm activities (inhibition zones up to 29 mm, biofilm inhibition up to 93.11%) are also notable, the exceptional potency against carbohydrate-hydrolyzing enzymes and butyrylcholinesterase—with IC_50_ values approaching those of acarbose (2.39–2.87 µg/mL) and rivastigmine (0.66 µg/mL)—suggests the highest translational potential in diabetes and Alzheimer’s disease management. The molecular docking results further support these findings by showing strong binding interactions of chlorogenic acid with lysosomal acid-α-glucosidase (docking score −6.96 kcal/mol) and butyrylcholinesterase (−6.54 kcal/mol). Collectively, these findings highlight carrot seeds as a valuable source of multifunctional phytochemicals and demonstrate that ethanol-modified supercritical fluid extraction is a promising strategy for developing natural bioactive preparations with pharmaceutical and nutraceutical applications, particularly for glycemic control and neuroprotection.

## Figures and Tables

**Table 1:** Effect of ethanol percentage as a co-solvent on the yield of extract from 4.0 g of dried carrot seeds under fixed supercritical fluid extraction conditions (40 °C, 250 bar, 25 min static time, and 40 min dynamic time). Extract quantity (mg) per 4.0 g dried carrot seeds. Results are represented as means ± SD from three independent extractions.

Temperature (°C)	Pressure (Bar)	Duration of Static Extraction (min)	Duration of Dynamic Extraction (min)	Modifier % (Ethanol)	Recovered Extract Amount (mg)
40	250	25	40	0	110 ± 0.2
40	250	25	40	5	134 ± 0.3
40	250	25	40	10	132 ± 0.2

**Table 2:** Phenolic compounds identified in carrot seed extracts obtained by supercritical CO_2_ extraction using 0% ethanol and 5% ethanol as co-solvents; RT: Retention time.

Compound Name	Ethanol Modifier (%)							
	0				5			
	Peak Number	RT (min)	Type	Conc. (µg/g)	Peak Number	RT (min)	Type	Conc. (µg/g)
Gallic acid	1	3.54	BV	706.91	1	3.54	BV	1279.27
Chlorogenic acid	2	4.26	MM	1464.18	2	4.14	VB	1541.24
Catechin	3	4.47	--	49.17	3	4.63	BB	51.76
Methyl gallate	4	5.67	BV	59.48	4	5.66	MM	38.06
Caffeic acid	5	5.91	VB	237.61	5	5.92	VB	279.01
Syringic acid	6	6.32	MM	537.27	6	6.32	BV	487.28
Rutin	7	6.69	--	0.00	7	6.73	VB	117.19
Ellagic acid	8	7.04	BV	80.90	8	7.24	BV	1186.05
Coumaric acid	9	8.74	BV	271.49	9	8.75	BV	336.86
Vanillin	10	9.34	BB	14.26	10	9.34	BB	23.33
Ferulic acid	11	9.83	BV	136.77	11	9.83	BV	149.41
Naringenin	12	10.46	MM	673.29	12	10.45	VB	988.09
Rosmarinic acid	13	11.89	BV	308.07	13	11.88	BV	397.52
Daidzein	14	16.02	BB	44.04	14	15.82	VB	146.79
Quercetin	15	17.66	MM	212.47	16	17.66	MM	258.48
Cinnamic acid	16	19.79	BB	71.31	17	19.78	BB	67.41
Kaempferol	17	20.99	MM	346.08	18	20.75	BV	65.92
Hesperetin	18	21.41	MM	16.85	19	21.42	VV	1513.68

**Table 3:** Effect of 5% ethanol as an extraction modifier on the antimicrobial activity of carrot seed extracts against selected microorganisms, as evaluated by inhibition zones, minimum inhibitory concentration (MIC), and minimum bactericidal concentration (MBC). Results are presented as mean ± standard deviation (SD). Within each row, different superscript letters denote statistically significant differences at *p* ≤ 0.05.

Examined Microorganisms	Inhibition Zones (mm)			MIC		MBC	
	Ethanol Modifier (%)		Positive Control (Gentamycin/Nystatin)	Ethanol Modifier (%)		Ethanol Modifier (%)	
	0	5		0	5	0	5
*Enterococcus faecalis*	15 ± 0.5 ^a^	19 ± 0.2 ^b^	20 ± 0.3 ^a^	62.5	15.62	250	31.25
*Staphylococcus aureus*	19 ± 0.2 ^a^	25 ± 0.9 ^b^	21 ± 0.2 ^a^	31.25	15.62	62.5	31.25
*Escherichia coli*	21 ± 0.5 ^a^	24 ± 1.0 ^b^	25 ± 0.8 ^a^	31.25	15.62	62.5	31.25
*Klebsiella pneumoniae*	25 ± 0.3 ^a^	26 ± 0.5 ^a^	25 ± 0.4 ^a^	15.62	15.62	62.5	31.25
*Candida albicans*	26 ± 0.4 ^a^	29 ± 0.3 ^a^	32 ± 0.1 ^a^	15.62	15.62	31.25	31.25
*Aspergillus niger*	0.0	0.0	34 ± 1.0 ^a^	--	--	--	--

**Table 4:** Impact of ethanol as an extraction modifier on the antibiofilm potential of carrot seed extracts. Within each row, different superscript letters denote significant differences (*p* ≤ 0.05).

Examined Microorganisms	Concentration (%)	Ethanol Modifier (%)	
		0	5
*Enterococcus faecalis*	25% MBC	46.60 ± 2.5 ^c^	76.90 ± 3.1 ^a^
	50% MBC	73.42 ± 2.0 ^b^	87.41 ± 2.8 ^a^
	75% MBC	89.84 ± 1.5 ^b^	93.11 ± 1.2 ^a^
*Staphylococcus aureus*	25% MBC	75.35 ± 2.1 ^a^	77.67 ± 2.0 ^a^
	50% MBC	85.82 ± 1.5 ^a^	87.12 ± 1.3 ^a^
	75% MBC	92.08 ± 1.2 ^a^	92.54 ± 1.0 ^a^
*Klebsiella pneumoniae*	25% MBC	68.86 ± 2.0 ^b^	70.34 ± 1.8 ^a^
	50% MBC	78.91 ± 1.5 ^b^	84.46 ± 2.1 ^a^
	75% MBC	88.36 ± 1.0 ^b^	91.00 ± 1.2 ^a^
*Escherichia coli*	25% MBC	38.21 ± 2.2 ^c^	74.68 ± 3.0 ^a^
	50% MBC	69.57 ± 1.8 ^b^	84.85 ± 2.5 ^a^
	75% MBC	83.43 ± 1.5 ^b^	92.91 ± 1.2 ^a^
*Candida albicans*	25% MBC	57.75 ± 2.0 ^b^	69.33 ± 2.1 ^a^
	50% MBC	84.40 ± 1.5 ^a^	86.17 ± 1.3 ^a^
	75% MBC	89.28 ± 1.2 ^a^	91.79 ± 1.0 ^a^

**Table 5:** In vitro α-amylase and α-glucosidase inhibitory potential of carrot seed extracts obtained using 0% and 5% ethanol.

Concentration (µg/mL)	Inhibition (%)					
	α-Amylase			α-Glucosidase		
	Acarbose	Ethanol Modifier (0%)	Ethanol Modifier (5%)	Acarbose	Ethanol Modifier (0%)	Ethanol Modifier (5%)
0.0	0.00 ± 0.00 ^a^	0.00 ± 0.00 ^a^	0.00 ± 0.00 ^a^	0.00 ± 0.00 ^a^	0.00 ± 0.00 ^a^	0.00 ± 0.00 ^a^
1.95	43.1 ± 0.8 ^a^	26.0 ± 2.0 ^c^	28.0 ± 2.1 ^b^	44.6 ± 0.7 ^a^	24.0 ± 1.5 ^c^	28.0 ± 2.0 ^b^
3.9	52.7 ± 1.0 ^a^	35.0 ± 3.0 ^c^	37.0 ± 3.2 ^b^	52.0 ± 0.8 ^a^	33.0 ± 2.0 ^b^	34.0 ± 2.5 ^b^
7.81	61.8 ± 1.2 ^a^	44.0 ± 3.5 ^c^	46.0 ± 3.0 ^b^	58.6 ± 1.0 ^a^	41.0 ± 2.5 ^b^	42.0 ± 2.7 ^b^
15.62	68.0 ± 1.5 ^a^	51.0 ± 3.0 ^c^	53.0 ± 2.5 ^b^	64.5 ± 1.2 ^a^	48.0 ± 2.0 ^b^	49.0 ± 2.2 ^b^
31.25	75.3 ± 1.0 ^a^	61.0 ± 2.5 ^c^	62.0 ± 2.0 ^b^	72.5 ± 1.1 ^a^	56.0 ± 2.0 ^b^	57.0 ± 2.0 ^b^
62.5	80.6 ± 1.2 ^a^	68.0 ± 2.0 ^b^	69.0 ± 2.0 ^b^	80.1 ± 1.0 ^a^	63.0 ± 1.8 ^b^	64.0 ± 2.0 ^b^
125	86.0 ± 0.8 ^a^	77.0 ± 1.5 ^b^	78.0 ± 1.2 ^b^	85.8 ± 0.7 ^a^	71.0 ± 2.0 ^b^	72.0 ± 1.5 ^b^
250	91.3 ± 0.7 ^a^	85.0 ± 1.0 ^b^	86.0 ± 1.0 ^b^	92.1 ± 0.8 ^a^	79.0 ± 1.5 ^b^	80.0 ± 1.2 ^b^
500	93.0 ± 0.5 ^a^	90.0 ± 0.7 ^b^	91.0 ± 0.6 ^b^	94.7 ± 0.6 ^a^	86.0 ± 1.0 ^b^	87.0 ± 0.8 ^b^
1000	97.4 ± 0.4 ^a^	94.0 ± 0.5 ^b^	95.0 ± 0.5 ^b^	97.5 ± 0.4 ^a^	92.0 ± 0.5 ^b^	93.0 ± 0.5 ^b^
IC_50_ (µg/mL)	2.39 ± 0.20	18.56 ± 1.2	7.74 ± 0.66	2.87 ± 0.25	25.08 ± 1.25	13.37 ± 0.5

Note: Different superscript letters denote significant differences (*p* ≤ 0.05).

**Table 6:** Ethanol-Dependent Butyrylcholinesterase Inhibition by Carrot Seed Extracts (0 and 5% ethanol). Values in the same row bearing different letters differ significantly (*p* < 0.05).

Concentration (µg/mL)	BChE Inhibition (%)		
	Rivastigmine	Ethanol Modifier (0%)	Ethanol Modifier (5%)
0	0.0 ± 0.0 ^a^	0.0 ± 0.0 ^a^	0.0 ± 0.0 ^a^
0.195	35.3 ± 0.8 ^a^	3.1 ± 2.0 ^c^	23.9 ± 2.1 ^b^
0.39	45.4 ± 1.0 ^a^	6.7 ± 2.5 ^c^	32.0 ± 2.5 ^b^
0.78	52.8 ± 1.2 ^a^	9.9 ± 3.0 ^c^	39.5 ± 3.0 ^b^
1.56	60.2 ± 1.3 ^a^	13.1 ± 3.5 ^c^	43.5 ± 3.0 ^b^
3.125	64.7 ± 1.5 ^a^	32.1 ± 3.0 ^b^	51.3 ± 2.5 ^b^
6.25	70.1 ± 1.2 ^a^	42.1 ± 3.0 ^b^	58.3 ± 2.5 ^b^
12.5	78.2 ± 1.0 ^a^	54.8 ± 2.5 ^b^	66.6 ± 2.0 ^b^
25	85.6 ± 1.0 ^a^	62.2 ± 2.0 ^b^	73.8 ± 1.5 ^b^
50	90.6 ± 0.8 ^a^	73.7 ± 1.5 ^b^	79.8 ± 1.5 ^b^
100	93.9 ± 0.7 ^a^	82.6 ± 1.5 ^b^	89.3 ± 1.0 ^b^
IC_50_ (µg/mL)	0.66	10.50	2.51

**Table 7:** Docking scores and binding energies of chlorogenic acid and vanillin with *Klebsiella pneumoniae* OmpK36 (PDB ID: 5O79).

Mol	S	rmsd_refine	E_conf	E_place	E_score1	E_refine	E_score2
Chlorogenic acid	−6.61821	1.1498349	−3.40435	−100.86	−13.2747	−32.3419	−6.61821
Chlorogenic acid	−6.44341	2.2405727	−9.82746	−80.4731	−12.1394	−33.3065	−6.44341
Chlorogenic acid	−6.41028	1.1861995	−5.48409	−82.2266	−11.7538	−31.8687	−6.41028
Chlorogenic acid	−6.20357	1.0129845	−9.63435	−105.651	−12.9024	−30.7988	−6.20357
Chlorogenic acid	−6.11751	1.7993412	−2.00471	−85.2333	−12.6259	−35.8241	−6.11751
Vanillin	−4.27697	0.87364501	−7.823109	−54.0547	−8.02798	−18.0459	−4.27697
Vanillin	−4.21902	2.6662476	−8.250064	−50.0085	−8.94046	−17.7634	−4.21902
Vanillin	−4.17443	0.74613839	−9.399827	−57.021	−8.71614	−14.6639	−4.17443
Vanillin	−4.12024	1.4292881	−7.558381	−55.126	−8.09572	−17.0856	−4.12024
Vanillin	−4.10692	1.310516	−7.581243	−64.457	−8.41731	−18.5478	−4.10692

**Table 8:** Docking scores and binding energies of chlorogenic acid and vanillin with human lysosomal acid α-glucosidase (PDB ID: 5NN5).

Mol	S	rmsd_refine	E_conf	E_place	E_score1	E_refine	E_score2
Chlorogenic acid	−6.96112	1.5436684	−0.9139	−68.5716	−11.6586	−40.7641	−6.96112
Chlorogenic acid	−6.88714	1.9445686	−1.48913	−91.4574	−12.2041	−38.5173	−6.88714
Chlorogenic acid	−6.78119	1.3087373	−5.80856	−108.973	−15.5163	−37.0431	−6.78119
Chlorogenic acid	−6.65382	1.2424115	−3.53608	−67.4245	−12.8214	−33.6445	−6.65382
Chlorogenic acid	−6.57293	2.1756837	−8.542692	−69.7038	−13.6518	−32.165	−6.57293
Vanillin	−4.77945	1.0974865	−9.928105	−51.3705	−8.68519	−1.1197	−4.77945
Vanillin	−4.50197	2.000922	−8.781432	−57.4811	−9.77935	−19.1247	−4.50197
Vanillin	−4.49624	2.0549743	−8.534404	−57.459	−9.18236	−17.1379	−4.49624
Vanillin	−4.48127	0.53130108	−8.101929	−73.7396	−9.55613	−17.8068	−4.48127
Vanillin	−4.36552	1.5313629	−8.928833	−71.5798	−8.76715	−16.9441	−4.36552

**Table 9:** Docking scores and binding energies of chlorogenic acid and vanillin with human butyrylcholinesterase (PDB ID: 4BDS).

Mol	S	rmsd_refine	E_conf	E_place	E_score1	E_refine	E_score2
Chlorogenic acid	−6.54409	1.9320935	−11.41547	−110.72	−10.8258	−34.9086	−6.54409
Chlorogenic acid	−6.47882	1.3184953	−3.985485	−100.486	−11.1266	−33.2043	−6.47882
Chlorogenic acid	−6.33181	1.5084401	−6.14806	−104.873	−11.5796	−29.8944	−6.33181
Chlorogenic acid	−6.33131	2.0202899	−3.696198	−72.0527	−11.8564	−31.9639	−6.33131
Chlorogenic acid	−6.27098	2.7554073	−4.590563	−67.9241	−10.7604	−30.7131	−6.27098
Vanillin	−4.67145	0.75694287	−8.031394	−50.0945	−8.3695	−19.9865	−4.67145
Vanillin	−4.66334	1.4726768	−10.03519	−54.3307	−9.52013	−20.688	−4.66334
Vanillin	−4.63552	0.84617698	−7.661886	−66.2767	−7.78153	−20.5703	−4.63552
Vanillin	−4.59382	2.8908732	−8.961206	−51.7885	−8.28553	−20.5751	−4.59382
Vanillin	−4.52074	2.5553885	−7.57086	−56.2738	−7.75886	−20.3514	−4.52074

**Table 10:** Molecular interactions of chlorogenic acid and vanillin with *Klebsiella pneumoniae* OmpK36 (PDB ID: 5O79).

Mol	Ligand	Receptor	Distance	Interaction	E (kcal/mol)
Chlorogenic Acid	O 2	NH1 ARG 75 (A)	3.03	H-acceptor	−1.3
	O 6	CE LYS 16 (A)	3.28	H-acceptor	−0.6
Vanillin	O 2	OD2 ASP 106 (A)	3.14	H-donor	−1.1
	O 3	NH1 ARG 37 (A)	3.04	H-acceptor	−1.7

**Table 11:** Molecular interactions of chlorogenic acid and vanillin with human lysosomal acid α-glucosidase (PDB ID: 5NN5).

Mol	Ligand	Receptor	Distance	Interaction	E (kcal/mol)
Chlorogenic Acid	O 3	O GLY 123 (A)	2.85	H-donor	−1.1
	O 4	N GLY 123 (A)	3.05	H-acceptor	−2.0
	6-ring	N ILE 98 (A)	3.82	pi-H	−0.5
Vanillin	O 2	O TRP 273 (A)	2.89	H-donor	−1.2

**Table 12:** Molecular interactions of chlorogenic acid and vanillin with human butyrylcholinesterase (PDB ID: 4BDS).

Mol	Ligand	Receptor	Interaction	Distance	E (kcal/mol)
Chlorogenic Acid	O 5	O ASN 228 (A)	H-donor	2.97	−1.8
	O 8	O PRO 230 (A)	H-donor	3.00	−0.8
	O 9	OE1 GLU 238 (A)	H-donor	2.87	−4.5
Vanillin	O 3	NH1 ARG 242 (A)	H-acceptor	3.32	−1.0
	O 3	NH2 ARG 242 (A)	H-acceptor	2.96	−0.7
	6-ring	CA SER 287 (A)	pi-H	3.87	−0.6

## Data Availability

The original contributions presented in this study are included in the article. Further inquiries can be directed to the corresponding author.
